# The Impact of the Perception of Primary Facial Emotions on Corticospinal Excitability

**DOI:** 10.3390/brainsci13091291

**Published:** 2023-09-06

**Authors:** Francesca Fiori, Andrea Ciricugno, Zaira Cattaneo, Chiara Ferrari

**Affiliations:** 1 Research Unit of Neurophysiology and Neuroengineering of Human-Technology Interaction (NeXTlab), Department of Medicine, Campus Bio-Medico University, 00128 Roma, Italy; f.fiori@unicampus.it; 2 Social Experimental Psychology Unit, IRCCS Mondino Foundation, 27100 Pavia, Italy; andrea.ciricugno@mondino.it (A.C.); chiara.ferrari@unipv.it (C.F.); 3 Department of Brain and Behavioral Sciences, University of Pavia, 27100 Pavia, Italy; 4 Department of Human and Social Sciences, University of Bergamo, 24129 Bergamo, Italy; 5 Department of Humanities, University of Pavia, 27100 Pavia, Italy

**Keywords:** TMS, motor-evoked potential, corticospinal excitability, emotion

## Abstract

The link between emotional experience and motor body responses has long been acknowledged. A well-established approach to exploring the effect of the perception of emotional stimuli on the motor system is measuring variations in the excitability of the corticospinal tract (CSE) through motor-evoked potentials (MEP) elicited via transcranial magnetic stimulation (TMS). Previous evidence has indicated a selective increase in MEP amplitude while participants view emotional stimuli, such as emotional facial expressions, compared to neutral cues. However, it is still not clear whether this effect depends on the specific emotional meaning conveyed by the stimulus. In the present study, we explored whether viewing faces expressing the primary emotions compared to faces with a neutral expression affects individuals’ CSE, measured using TMS-elicited MEPs. Specifically, we elicited MEPs from the left motor cortex (M1) while participants passively viewed the same faces expressing either anger, fear, disgust, happiness, sadness, surprise, and no emotion (in different blocks). We found that the observation of fearful, angry, disgusted, and happy facial expressions was associated with a significant increase in the MEPs’ amplitude compared to neutral facial expressions, with a comparable enhancement in the CSE occurring across these emotions. In turn, viewing sad and surprised faces did not modulate the CSE. Overall, our findings suggest that only facial expressions that signal (real or potential) danger or a rewarding stimulus, but not emotional facial expressions per se, are capable of activating action-related mechanisms.

## 1. Introduction

The ability to recognize emotional expressions is critical for survival and plays a pivotal role in successful social interactions. Converging evidence has suggested that the processing of emotional faces triggers the activation of widespread brain regions (for a review, see [1]), including motor areas such as the supplementary motor, premotor, and primary motor cortex [2,3]. The activation of the motor system during the perception of emotional faces has been interpreted as reflecting mechanisms of the preparation of adaptive motor responses, such as the fight/flight response [4,5], consistently with the long-held view that emotions prime the human body for action [6,7,8].

More direct evidence that emotional faces prime motor responses has come from studies employing transcranial magnetic stimulation (TMS), which allows for quantifying corticospinal excitability (CSE) by the magnitude of elicited motor-evoked potential (MEP) over the primary motor cortex [9]. MEPs have been widely used to assess whether and how viewing emotional stimuli affects the excitability of the primary motor cortex [10,11]. Consistent evidence has suggested that viewing highly arousing emotional scenes is associated with an increased CSE compared to low-arousing emotional or neutral scenes [10,12,13,14]. The effective modulation of the CSE has also been reported for stimuli such as emotional bodies and faces [15,16,17,18,19,20,21], with the pattern of effects on MEPs also depending on the specific timing of the stimulation, reflecting different stages of elaboration by the sensorimotor system [16].

Studies that have assessed changes in MEPs due to viewing emotional faces have mostly tested negative (anger or fear) vs. neutral or happy expressions. For instance, Schutter and colleagues [21] and Borgomaneri et al. [18] presented participants with happy, fearful, and neutral facial expressions while delivering TMS pulses to the motor cortex and recording MEPs from the contralateral hand. The findings from both studies converged in showing that the MEPs’ amplitudes were bigger when registered while watching fearful compared to neutral facial expressions. The effect of fearful faces on the CSE was also replicated by Ferrari and colleagues [19]. With regard to happy vs. neutral faces, Borgomaneri et al. [18] reported an increment in MEP amplitudes for happy compared to neutral expressions, while Schutter and colleagues [21] found no significant differences between these conditions. Moreover, Salvia et al. [22] found a CSE enhancement when participants viewed individuals performing dynamic facial actions (e.g., such as opening the mouth) in an angry compared to neutral way. Finally, Vicario et al. [23], who assessed the MEPs elicited by applying TMS over the left M1 during the viewing of disgusted vs. happy vs. neutral expressions, failed to report any significant modulation of the MEPs as a function of the emotional content conveyed by the faces.

The overall existing evidence suggests that perceiving emotional faces somehow impacts the CSE. However, due to the limited number of studies and the methodological differences within them, it is still unknown whether the modulation of the CSE in response to emotional facial expressions varies as a function of the specific emotional content/meaning conveyed by the face or reflects differences in emotional valence (positive vs. negative). In this study, we aimed to provide a systematic investigation of the emotional-related modulation of the CSE (assessed using TMS-induced MEP) by presenting, within the same experimental session, facial expressions of all the primary emotions, namely disgust, fear, anger, sadness, happiness, and surprise, in addition to neutral facial expressions. Based on previous findings [18,19,21,22], we expect to find that the perception of fearful and angry faces increases the CSE in comparison to neutral faces. In turn, we expect to not find an effective emotional modulation of the CSE in response to happy and disgusted facial expressions, consistent with previous evidence [21,23]; but see [18]. No work has so far been conducted with sad and surprised faces; therefore, we do not have specific hypotheses on whether the perception of these two emotional faces might impact the CSE. Furthermore, since the perceived arousal of the stimuli has been identified as a critical aspect in determining emotion-related CSE modulation, in particular for emotional scenes [10,13], following the recording of MEPs, our participants rated the arousal of the faces. The face arousal ratings were then correlated with the MEPs’ amplitudes to explore the relationship between these two variables.

## 2. Methods

### 2.1. Participants

Twenty-two university students took part in the TMS experiment (four males; mean age = 24.1, SD = 2.9 years). All the participants were right-handed [24] and had normal or correct-to-normal vision. The sample size was defined based on previous studies [18] and confirmed by a power analysis conducted using the G-Power 3.1 software. The analysis indicated that a sample size of 19 individuals was required to obtain a 90% power at a significance threshold of 0.05 two-tailed, with an expected large effect size of *dz* = 0.78 based on data from a prior TMS study [18]. Before the TMS experiment, each participant filled in a questionnaire to evaluate their compatibility with TMS (translated from Rossi et al. [25]). Written informed consent was obtained from all the participants. The study was conducted according to the guidelines of the Declaration of Helsinki, and approved by the Institutional Review Board of the University of Pavia.

### 2.2. Stimuli

**Figure 1:**
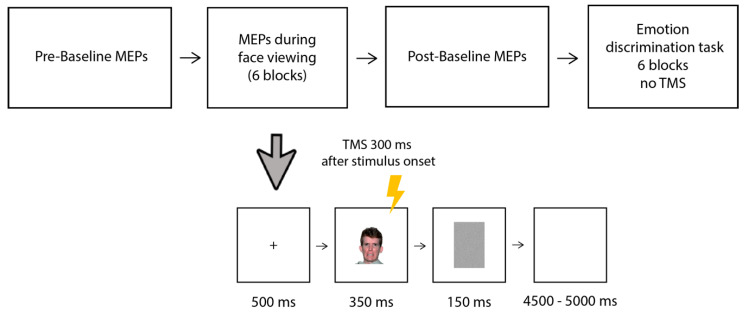
The experimental paradigm (upper panel) and timeline of an experimental trial (lower panel). MEPs were recorded from the right FDI (following TMS over the left M1) during the viewing of emotional and neutral faces; two baseline blocks (with no stimuli presented) were performed before and after the main MEP session (15 MEPs were recorded in the baseline-pre and 15 in the baseline-post). The main experiment consisted of 6 experimental blocks: in each block, 40 faces were presented showing either an emotional (20 trials) or neutral (20 trials) facial expression. In each block, the emotional faces expressed the same emotion (one of the six basic emotions). The MEPs session was followed by a discrimination task without TMS, in which participants were presented again with the same 6 blocks viewed before and had to discriminate the emotional from neutral faces by left/right key pressing. The face depicted in Figure 1 was obtained from the NimStim database [26].

The visual stimuli consisted of 140 images obtained from the NimStim database [26], see Figure 1) depicting young Caucasian individuals. The same 20 individuals (10 males and 10 females) were depicted while expressing the six primary emotions (anger, fear, disgust, sadness, happiness, and surprise), and an additional neutral/non-emotional expression in a frontal pose.

### 2.3. Electromyographic Recordings and Transcranial Magnetic Stimulation (TMS)

During the experiment, the participants were seated with support for their right arm and hand. MEPs were recorded from the right dorsal interosseous (FDI) muscle using a pair of disposable surface electrodes placed in a belly tendon montage. The reference electrode was placed on the joint between the first and second phalanx, and the ground electrode on the wrist. The electromyographic signal (EMG) was acquired with a CED Power 1401 electromyograph controlled with the software Signal 3.13 (Cambridge Electronic Devices, Cambridge, UK). The EMG was amplified with a Digitimer D360 amplifier (Digitimer Ltd., Welwyn Garden City, Hertfordshire, UK) and filtered at 20 Hz and 2 kHz. Traces were then digitized at a sampling rate of 5 kHz and stored for offline analysis.

The optimal scalp position for inducing MEPs in the right FDI muscle was found by moving the coil in steps of 1 cm over the M1 until the largest MEPs were found. Then, the position was marked with a felt pen on a tight-fitting swimming cap worn by the participants. The coil was held tangential to the scalp with the handle pointing backward and laterally at a ~45° angle away from the midline [27,28]. Then, we determined the resting Motor Threshold (rMT), which is defined as the minimal TMS intensity required to elicit MEPs of at least a 50 µV amplitude from the contralateral FDI muscle in at least 5 out of 10 consecutive trials [29]. Single-pulse TMS was delivered using a 70 mm figure-of-eight coil connected to a Magstim Rapid^2^ stimulator, with the intensity of stimulation set at 120% of the individual rMT (mean of the maximum stimulator output = 60.3; SD = 10.2). The TMS pulses were delivered with an inter-pulse interval of ~8–10 s, a long inter-trial interval that has been demonstrated not to induce any change in cortical excitability [30].

### 2.4. Procedure

The participants were comfortably seated in a quiet and half-light room at an approximate distance of 60 cm from a 19′ computer screen. After the electrodes montage, the identification of the optimal scalp position for inducing MEPs, and the assessment of the individuals’ rMT, the participants underwent a short baseline pre-session, in which 15 MEPs were collected from the right FDI (as in the main MEP session). During the baseline pre-session (lasting approximately 2.5 min), the participants kept their eyes open and passively looked at the wall in front of them. TMS pulses were delivered over the left M1 at a random interval ranging from 8 to 10 s.

Following the baseline session, the participants were presented with the emotional faces. The experiment included the presentation of a total of 240 images depicting emotional and neutral faces, organized into six blocks (i.e., 40 mages per block). Each block consisted of the presentation of 20 emotional faces belonging to one of the six primary emotions and 20 faces with a neutral expression (i.e., each of the 20 different faces was presented once with an emotional expression and once with a neutral expression within each block). This emotion-blocked design has been found to facilitate a sort of emotional ‘‘contagion’’ throughout the prolonged exposition of the same emotional content and helps participants to focus on a particular emotion [31,32].

The timeline of the experimental trial is shown in Figure 1. Each trial started with a black fixation cross on a white background (500 ms), followed by the presentation in the center of the screen of the face (350 ms) and a gray mask (150 ms); then, a blank screen was presented for a random duration ranging from 4500 to 5000 ms. The participants were told to pay attention to the faces because, at the end of the session, they would be required to answer some questions about them. Single-pulse TMS was delivered to the left motor cortex (M1) 300 ms after the onset of the face, in line with previous studies [19,21]. Within each block, the trials were presented in a random order and the order of the blocks was counterbalanced among the participants. Each block took approximately 6 min; the participants were allowed short breaks (of around 2 min) between the blocks. After performing all 6 blocks, the participants underwent a second baseline post-session, in which an additional 15 MEPs were collected following the same procedure as that in the baseline pre-session.

After the MEP session, the participants performed an emotion discrimination task (with no TMS) to ensure that they could properly discriminate between the neutral and emotional expressions within each block. The same 6 blocks of the MEP session were presented, and the participants had to indicate by a left/right key press using their right hand whether the face was expressing an emotion or was neutral (with the association of left/right neutral/emotion counterbalanced across the subjects). Prior to each block, the participants were informed about the specific emotion they had to discriminate from neutral in the block. The trial structure was the same as that in the MEP session, but the blank screen following the mask remained visible only until the participants responded (to make the task faster) and no TMS was delivered. The participants were encouraged to respond as accurately and quickly as possible.

The software E-prime 2.0 (Psychology Software Tools, Inc., Pittsburgh, PA, USA) was used for the stimuli presentation, TMS triggering, and data recording. The whole experimental session lasted approximately 1 h and 45 min, including instructions and debriefing.

All the 140 faces used in the experiment were then evaluated by the same participants in terms of arousal in an online rating (Qualtrics Survey, Provo, UT, USA) performed a few days after the MEP experiment (range 3–7 days). The faces were presented in a random order using the Self-Assessment Manikin [33] combined with a 1–9 Likert scale: 1 = minimally aroused/calm and 9 = highly aroused/excited. The faces remained on the screen until the participants responded.

## 3. Data Analyses and Results

### 3.1. Data Analyses

The MEP amplitudes were processed offline and measured as the peak-to-peak (in mV). The mean rectified signal of the EMG background activity, 100 ms prior to the TMS pulses, was calculated, and MEPs with preceding EMG activity deviating from the mean rectified signal by >2.5 SD were removed from the analysis (resulting in the removal of 3% of the overall number of collected MEPs). To assess whether the prolonged stimulation determined changes in the CSE over time, a preliminary pairwise *t*-test was carried out to compare the MEP amplitudes across the two baseline blocks (pre- and post-session). Furthermore, to ensure that the possible modulation observed during the emotional faces presentation did not depend on spontaneous CSE oscillations or that no learning effect occurred over the experiment, we ran a repeated-measures ANOVA to compare the raw MEP amplitudes evoked in response to the neutral faces across the six blocks.

Following these preliminary analyses, for each participant, the raw MEP amplitudes recorded during the emotional faces presentation were normalized (divided) by the MEP amplitudes recorded during the neutral faces presentation of the same block (i.e., MEP emotional faces/MEP neutral faces included in the same block). To test whether the perception of each of the six basic emotions was associated with a facilitation or inhibition of the CSE as compared to the neutral face condition, a one-sample *t*-test against 1 was performed for each block. Furthermore, the normalized MEP amplitudes that were statistically different from 1 were submitted to a univariate ANOVA with emotion as a factor to allow for a direct comparison among emotions.

Moreover, to ensure that the participants could correctly discriminate between the emotional and neutral faces, the accuracy rates of the emotional discrimination task (without TMS) were analyzed by the means of a repeated-measures ANOVA with emotion (anger, fear, disgust, sadness, happiness, and surprise) as a within-subjects factor. Moreover, another repeated-measures ANOVA with emotion (anger, fear, disgust, sadness, happiness, surprise, and neutral) as a within-subjects factor was conducted on the face arousal ratings that the participants provided in the online task. Finally, to test whether CSE modulation was associated with perceived arousal and recognition rates, we correlated the (raw) MEP amplitudes recorded in response to the emotional and neutral faces with the perceived arousal and accuracy rates of each face expression (Pearson correlation).

Partial *η*^2^ *(η_p_*^2^*)* was computed as a measure of effect size for the significant ANOVA main effect and interactions, whereas Cohen’s d indices were computed for significant *t*-tests or post hoc comparisons and the Greenhouse–Geisser correction was applied when the sphericity assumption was violated.

### 3.2. Results

#### 3.2.1. MEP

The MEP amplitudes across the two baseline blocks were comparable, *t*(21) = 0.568, *p* = 0.58, indicating that TMS, per se, did not change the CSE over time. Similarly, the raw MEP amplitudes evoked in response to the neutral faces (baseline stimuli) did not differ across the six blocks, *F*(5, 105) = 0.812, *p* = 0.54, *η_p_*^2^ = 0.04. This result indicates that there were no significant spontaneous fluctuations in the CSE throughout the experiment and no learning effect due to repeated exposure to neutral faces occurred over the experiment.

**Figure 2:**
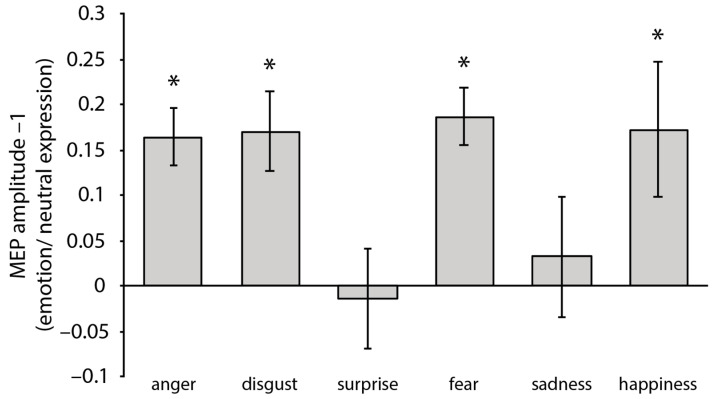
MEPs amplitude (mV) for each emotion, normalized to neutral expression (i.e., MEP emotional faces/MEP neutral faces included in the same block). Positive values indicate that higher MEP amplitudes were observed in response to emotional compared to neutral faces (and vice versa for negative values). Error bars represent ± SEM. Asterisks indicate a significant difference in MEPs amplitudes in response to emotional compared to neutral expressions.

One-sample *t*-tests against 1 of the normalized MEP amplitudes (i.e., MEP emotional faces/MEP neutral faces included in the same block) computed for each emotion/block showed a significant increase in the MEP amplitudes in response to the angry faces, *t*(21) = 5.25, *p* < 0.001, *d* = 1.12, the fearful faces, *t*(21) = 5.84, *p* < 0.001, *d* = 1.25, the disgusted faces, *t*(21) = 3.85, *p* = 0.010, *d* = 0.82, and the happy faces, *t*(21) = 2.32, *p* = 0.030, *d* = 0.49 (see Figure 2). In turn, perceiving the sad faces, *t*(21) = 0.48, *p* = 0.63, and surprised faces, *t*(21) = 0.25, *p* = 0.81, did not significantly increase the MEP amplitudes. A repeated-measures ANOVA with emotion (anger, disgust, fear, and happiness; i.e., the only emotions modulating the CSE as assessed by the one-sample *t*-tests) as a within-subjects factor did not reveal any significant difference in the MEPs’ amplitude *F*(2.116, 44.443) = 0.35, *p* = 0.97 (Greenhouse–Geisser correction applied).

#### 3.2.2. Emotion Discrimination Task (No TMS)

The mean recognition accuracy (see Table 1) collected in the behavioral emotion discrimination task was, overall, 90.38% (SD = 10.68), indicating that the participants could successfully discriminate the emotional from the neutral facial expressions. The ANOVA revealed a significant effect of emotion, *F*(5105) = 6.85, *p* < 0.001, *η_p_*^2^ = 0.25: post hoc comparisons (Bonferroni–Holm correction applied) indicated that the participants performed significantly better with the angry faces, *t*(21) = 4.935, *p* < 0.001, *d* = 0.77, disgusted faces, *t*(21) = 4.825, *p* < 0.001, *d* = 0.76, fearful faces, *t*(21) = 3.033, *p* = 0.037, *d* = 0.48, and happy faces, *t*(21) = 4.273, *p* < 0.001, *d* = 0.67 compared to the sad faces. No other comparisons reached significance (all *t* < 2.84, *ps* > 0.06).

#### 3.2.3. Online Ratings (No TMS)

Table 2 shows the mean ratings (and SD) of the perceived arousal for the neutral and emotional expressions. The ANOVA revealed a significant effect of emotion, *F*(2.73, 57.322) = 65.114, *p* < 0.001, *η_p_*^2^ = 0.76. Post hoc comparisons (Bonferroni–Holm correction applied) showed that all the emotional faces were rated as more arousing than the neutral faces (all *ps* < 0.001, all *d* > 0.77). Among the emotional faces, the angry faces received the highest arousal ratings, followed by the disgusted, fearful, surprised, sad, and happy faces (least arousing). Specifically, the angry faces were perceived as more arousing than all the other emotional expressions (all *ps* < 0.007, all *ds* > 0.55), with the exception of disgust, *t*(21) = 1.035, *p* = 0.57. The disgusted faces were perceived as similarly arousing to the fearful faces *t*(21) = 2.216, *p* = 0.09, but more arousing than the other emotions (all *ps* < 0.007, all *ds* > 0.56). The fearful faces did not differ from the surprised faces, *t*(21) = 1.124, *p* = 0.53, but received higher arousal ratings compared to all the other emotions (all *ps* < 0.003, all *ds* > 0.61). The happy faces were perceived as the least arousing emotional expressions (lower than all the other emotions, all *ps* < 0.001, all *ds* > 0.77), and sadness did not differ from surprise *t*(21) = 2.521, *p* = 0.052.

### 3.3. Correlational Analyses

To test whether the level of perceived arousal might have influenced the observed CSE modulation, we conducted correlational analyses. An item-based Pearson correlational analysis between the MEPs’ amplitudes and their perceived arousal for the emotional faces was performed, resulting in not being significant, *r*(118) = 0.114, *p* = 0.214. The same analysis was also conducted for each expressed emotion separately; however, no significant correlations were revealed, all *ps* > 0.162.

Furthermore, we tested whether the recognizability of the emotional expressions might have been linked to CSE modulation by conducting an item-based Pearson correlational analysis between the (raw) MEP amplitudes and accuracy rates of each face. The analysis revealed a significant correlation between the two variables, *r*(140) = 0.40, *p* < 0.001. The same analysis conducted for each expressed emotion separately, in turn, showed no significant correlations (all *ps* > 0.07).

## 4. Discussion

In this study, we assessed the CSE modulation associated with viewing faces expressing the six primary emotions (i.e., fear, disgust, anger, happiness, surprise, and sadness). We found that observing faces expressing anger, disgust, fear, and happiness facilitated the CSE (with no differences across the four emotions), as compared to viewing the same faces showing neutral expressions. In turn, the observation of sad and surprised faces did not modulate the CSE compared to viewing neutral faces. Furthermore, our correlational analyses revealed that MEP modulations in response to emotional faces were not related to face arousal, but rather to the level of recognizability of the emotion conveyed by the face.

### 4.1. Fear and Anger

The increase in the CSE in response to the fearful faces was consistent with the seminal study of Schutter et colleagues [21] and more recent studies [18,19] that have found higher MEP amplitudes when observing fearful compared to neutral faces. We found a similar CSE enhancement in response to the angry faces, replicating the results of Salvia and colleagues [22], who presented participants with videos displaying individuals performing facial actions (e.g., such as opening the mouth) in an angry compared to neutral way.

### 4.2. Disgust

Our study also revealed an increase in MEP amplitude when viewing the disgusted faces as compared to the faces with neutral expressions. Facial expressions of disgust have previously been found to suppress M1 cortico-hypoglossal output, but not to affect the CSE [23]. However, it is worth noting that, in Vicario and colleagues [23], MEPs were measured from the extensor carpi radialis with TMS pulses delivered at random times ranging between 1100 and 1400 ms after the onset of the presentation of the face, a paradigm very different from that used in our study. The modulation of the CSE in response to the disgusted faces revealed here was consistent with previous evidence indicating that, from an evolutionary perspective, angry, fearful, and disgusted faces signal (potential or real) danger in the environment and are therefore particularly effective in priming the body for action. Accordingly, threat-related expressions elicit the activation of specific (defensive) brain circuits devoted to attention and action preparation [34], and, consequently, might trigger action tendencies more than other emotional stimuli. Therefore, the motor facilitation induced by the perception of fearful, angry, and disgusted faces observed in our study might reflect the augmented need for an action triggered by signals of danger.

### 4.3. Happiness

Critically, we found that the perception of the faces expressing happiness modulated the CSE and did it to the same extent as threat-related facial expressions. This finding fits well with the evolutionary theories [7,35] and behavioral studies suggesting that motor preparation is also observed in response to rewarding, positive stimuli, facilitating motor responses [36,37]. Indeed, although at a different timing (150 ms after the stimulus onset) and with a paradigm slightly different from the one used here (in which the participants were required to recognize the emotion while registering the MEPs), Borgomaneri et al. [18] found an increase in the CSE in response to happy vs. neutral facial expressions (in addition to fearful vs. neutral faces). Still, other evidence has failed to report an effect of happy faces on recorded MEPs [18,21] when elicited 300 ms from the face onset (like in our paradigm). However, it should be noted that in our study (differently from Schutter et al. and Borgomaneri et al. [18,21]), we employed an emotion-blocked design, in which the participants saw each emotion at a time (+neutral faces). This emotion-blocked design has been found to facilitate emotional contagion, helping participants to focus on a particular emotion [31,32], and this might have magnified the impact of happy facial emotional expressions on the CSE in our study. Indeed, the way different emotions are presented (blocked or intermixed) has been found to modulate MEP effects in prior studies [20]. Future studies should systematically investigate the effect of context on emotion-related CSE modulation, both in terms of design, instructions, or combining different stimuli.

### 4.4. Sadness and Surprise

We found a lack of CSE modulation in response to the sad expressions. Although we are the first to assess the impact of the perception of sad emotional facial expressions on the CSE, our result was consistent with prior studies, in which participants listened to sad vs. neutral music [38]. Nonetheless, self-induced sadness may be effective in modulating MEPs, as suggested by an earlier study by Tormos et al. [39]. In this study, the authors asked participants to think back to an event in their past that had induced sadness and found that self-induced sadness facilitated the MEPs’ amplitudes elicited by both the right and left M1. We can hypothesize that contemplating sad personal life events may have a more pronounced effect on the CSE compared to simply viewing the sad expression of a stranger. Whether self-induced emotions (also beyond sadness) might be capable of magnifying (or modifying) the effect that the perception of others’ emotions has on the CSE is an interesting issue that further studies might address. The limited evidence available on this has shown that self-induced vs. “perceived in others” emotions might have different effects on the CSE, depending on the specific emotion considered. Indeed, differently from sadness that, as mentioned before, enhances the MEPs’ amplitudes elicited by both right and left M1, self-induced happiness facilitates the MEPs evoked by right-hemispheric TMS (in line with the CSE modulation of the perception of happy faces, Borgomaneri et al. [18]), but decreases the amplitude of those evoked by left-hemispheric TMS [40].

With regard to surprised faces, to our best knowledge, little is known about the facial expression of surprise, and our study is the first to investigate the modulation of the CSE in response to surprised faces, demonstrating that the viewing of surprised facial expressions has no effect on the CSE. Surprise has been associated with novelty and the perception of surprised facial expressions in others may be related to the detection or evaluation of novel stimuli in the environment [41]. At a neurophysiological level, the lack of a modulation of the CSE, as compared to that with the viewing of neutral facial expressions, may be related to the sense of uncertainty conveyed by the emotion itself, with the organism “waiting” for additional information [42]. It is possible that delivering TMS at different times (later) from the onset of the stimulus might produce different results.

### 4.5. General Discussion

In line with the idea that emotions prime the body for action [6,7,8], our findings, overall, indicate that the modulation of the CSE due to viewing facial expressions is emotion-specific, possibly reflecting the response preparation to the peculiar meaning of an emotional cue. The effect of emotional faces on the CSE is a result of the complex interplay between various mechanisms occurring at different time points [43,44]. Indeed, by combining our findings with previous evidence, it is possible to observe that the CSE shows an early enhancement (150 ms after the face onset) in response to happy and fearful (vs. neutral) faces, possibly reflecting a first rapid response to emotional stimuli [18]. A second enhancement is visible at 300 ms after the face onset [21], but see [18], and our findings showed that this latter effect is specific to some emotional facial expressions that are more linked to an augmented need for action. At the speculative level, we might hypothesize that the different CSE responses to emotional faces reflect distinct stages of elaboration of the emotional material by the sensorimotor system, with the first CSE response possibly representing a low-level analysis of the stimuli (likely tapping into the activation of the subcortical regions) and the second CSE response representing the result of higher-order processing involving more extensive cortical pathways.

A critical result revealed by our study was that emotion-related CSE modulation does not simply reflect the perceived arousal (intensity) of an emotional expression. Indeed, the happy faces, which were rated as the least arousing among the emotional expressions (lower than all the other emotions), effectively modulated the MEPs’ amplitudes, while the surprised and sad faces were perceived as highly/moderated arousing (e.g., more arousing than the happy faces), but did not produce any significant modulation on the MEPs. Accordingly, our correlational analysis between the CSE modulation and arousal evaluations was not significant. Although previous studies have identified arousal as one key factor in driving the emotion modulation of the CSE [10,12,13], our data suggest that this is not the case for the CSE modulation specifically elicited by emotional facial expressions. It is worth noting that the sad faces we employed were rated as particularly arousing, although usually, sad stimuli are evaluated as low-level arousing [45], an effect that might have depended on our selection of stimuli that exaggeratedly displayed sadness, more resembling despair than genuine sadness.

In turn, our study revealed a relationship between MEP modulation and the recognizability of emotional faces (the accuracy at which the emotional faces were discriminated from the neutral ones in the emotion discrimination task). Indeed, although all the emotional expressions were, overall, recognized at a high level of accuracy (above 85%), the more participants were able to recognize them, the bigger the MEP modulation was, strengthening the idea that the modulation of MEP amplitudes is directly linked to the recognition of the specific emotional content conveyed by a face.

In our study, we intentionally restricted our investigation to the six primary emotions, but future investigations might investigate the impact of secondary emotions on the CSE, such as combinations of basic emotions, complex emotions, or social emotions. A few studies have shown that both the empathetic responses to pain [46] and the induction of feelings associated with social rejection [47] can modulate the CSE. This suggests that the effect of emotion on the CSE might not be limited to primary emotions, but it is also plausible that the modulation of the CSE induced by secondary emotions may display an even greater variability, possibly due to the influence of different cultural contexts that favor diverse interpretations of the same emotional stimuli.

Furthermore, the literature consistently suggests that the gender of participants affects their ability to recognize affective facial expressions, with women usually outperforming men [48,49,50]. We could not explore the impact of the participants’ gender on our behavioral and neurophysiological data, because our sample was highly imbalanced in terms of its male-to-female ratio (18 females vs. 4 males). However, whether the advantage shown by women over men in the behavioral indexes of emotion recognition is paralleled by a higher increment in the CSE in response to emotional faces is an issue that deserves further investigation. Furthermore, it would be interesting to investigate whether the gender of the faces (or the interaction between the gender of the participants and the gender of the faces) might play a role in eliciting different CSE responses.

Finally, it is important to note that the study of the effect of the perception of the emotional facial expressions on the CSE was characterized by high heterogeneity in terms of our designs and methodological choices, including the timing of the TMS stimulation (150 ms, 300 ms vs. 1100 ms after stimulus onset), the site of the TMS stimulation (left vs. right M1), the muscle from which the MEPs are recorded (e.g., FDI, vs. abductor pollicis brevis, APB), and the task performed by the participants during the MEP registrations (e.g., emotion recognition vs. passive viewing). Indeed, as already discussed before, we assessed the CSE at 300 ms after the stimulus onset, in line with previous studies [21], while Borgomaneri and colleagues [18] delivered TMS both at 150 ms and 300 ms after the presentation of emotional faces. Furthermore, we registered MEPs from the FDI, which is an extensor muscle, but other hand muscles, such as the flexor muscles (e.g., APB), might be used to register TMS-elicited MEPs. Different hand muscles have been linked to different approach/withdrawal-related responses, for instance, FDI plays a relevant role in approach movements and APB in withdrawal-related behavior [40,51]. Therefore, it is possible that delivering pulses at different time points or using different hand or body muscles to record EMG responses lead to different outcomes [18,23]. Moreover, we focused on the excitability of the hand motor representations in the left hemisphere, in line with previous evidence [19,20,21]. Although a similar MEP increment in response to the fearful (vs. neutral) facial expressions was found both in the right and left hemispheres [19], it is possible that emotion-specific modulations may occur differently in the right hemisphere, which is specialized for emotional processing (e.g., [18,52]; but see [53]). Future studies are needed to clarify these issues.

In summary, our findings indicate that, at the neurophysiological level, only when we are presented with faces that may convey the message of a possible threatening stimulus in the environment (i.e., fear, anger, and disgust) or a positive and rewarding stimulus is the body primed for action by alerting the organism, as soon as 300 ms from the appearance of the stimulus.

## Figures and Tables

**Table 1:** Mean (SD) of recognition accuracy of emotional faces vs. neutral faces.

	Anger	Disgust	Surprise	Fear	Sadness	Happiness
Recognition Accuracy %	93.05 (9.60)	92.86 (8.48)	89.59 (11.56)	89.91 (9.72)	84.91 (12.37)	91.95 (10.76)

**Table 2:** Mean (SD) of arousal ratings as a function of the emotion expressed by the faces.

	Anger	Disgust	Surprise	Fear	Sadness	Happiness	Neutral
Arousal	6.65 (1.49)	6.38(1.51)	5.49 (1.50)	5.79 (1.48)	4.82 (1.49)	3.67 (2.14)	2.45 (1.31)

## Data Availability

Deidentified data for all experiments are available on Zenodo.org (accessed on 3 September 2023).
